# 2′-deoxyinosine ameliorates multiple sclerosis through modulating Th1/Th2 and Th17/Treg cell balance with potential involvement of AMPK phosphorylation

**DOI:** 10.3389/fphar.2026.1837446

**Published:** 2026-09-17

**Authors:** Daqing Wang, Jiaxuan Zhu, Zhuo Wang, Zijing Guo, Yichun Zhu, Chunyan He

**Affiliations:** 1 Department of Clinical Laboratory, Suzhou TCM Hospital Affiliated to Nanjing University of Chinese Medicine, Suzhou, Jiangsu, China; 2 Department of Clinical Laboratory, Xuchang Central Hospital, Xuchang, Henan, China; 3 Department of Clinical Laboratory, Kunshan Hospital of Chinese Medicine, Affiliated Hospital of Yangzhou University, Kunshan, China

**Keywords:** AMPK, deoxyinosine, immune imbalance, inflammatory process, multiple sclerosis

## Abstract

**Background:**

Multiple sclerosis (MS) is an autoimmune and neurodegenerative disease characterized by T lymphocyte infiltration and demyelination that can lead to severe disability without treatment. However, effective treatment for MS remains an unmet need. 2′-deoxyinosine (2′-DI), an endogenous nucleoside, has been implicated in tumorigenesis and immune regulation. This study was performed to investigate whether prophylactic 2′-DI intervention could prevent MS progression and elucidate its action mechanism.

**Methods:**

2′-DI was administered to experimental autoimmune encephalomyelitis mice in vivo and cultured peripheral blood samples from MS patients in vitro, followed by pathological analysis, body weight monitoring, and disease severity assessment. Furthermore, RNA sequencing and serum metabolomics were conducted to explore the underlying regulatory mechanism of 2′-DI.

**Results:**

Compared with healthy controls, MS patients exhibited significantly reduced 2′-DI levels in peripheral blood. Prophylactic administration of 2′-DI could prevent the progression of MS and mitigate inflammatory infiltration in the EAE mouse model. Immunomodulatory studies revealed that 2'-DI could decrease Th1 and Th17 cells, whereas increase Treg and Th2 cells. Moreover, 2′-DI could also promote the expression of anti-inflammatory cytokines and transcription factors while inhibiting the expression of pro-inflammatory cytokines and chemokines. Notably, 2′-DI intervention increased AMPK phosphorylation status, suggesting a potential association between elevated AMPK phosphorylation and the 2′-DI-mediated reversal of Th1/Th2 and Th17/Treg cell imbalance.

**Conclusion:**

2′-DI may serve as a promising prophylactic candidate for MS by inhibiting central neuroinflammation and correcting peripheral and central Th1/Th2 and Th17/Treg immune imbalance, with its immunomodulatory effects potentially involving AMPK phosphorylation upregulation, thereby alleviating neuroinflammation and neurodegeneration.

## 1 Introduction

Multiple sclerosis (MS), a neuroinflammatory disease of the central nervous system (CNS) characterized by T lymphocyte infiltration, demyelination, and neuronal injury, is the leading cause of nontraumatic disability among young adults aged 18–40 years (Jakimovski et al., 2024; Kong et al., 2016; Wu et al., 2015). According to statistics from the World Health Organization, there are approximately 2.8 million people with MS worldwide. Notably, the disease exhibits a striking geographic variation in prevalence, with the highest rates in Europe and America (111–300 cases per 100,000) and the lowest in Africa and the Western Pacific region (5 per 100,000) (Jakimovski et al., 2024; GBD, 2016 Multiple Sclerosis Collaborators, 2019). Given this substantial global disease burden and uneven distribution of affected populations, the development of novel preventive and therapeutic strategies for MS and identification of sensitive diagnostic biomarkers are therefore highly warranted.

In the acute phase of MS, glucocorticoids (GCs) are mainly used to reduce symptoms, improve the degree of disability, shorten the disease course, and prevent complications (Wang et al., 2020). However, long-term treatment is associated with a high risk of severe toxic side effects, poor therapeutic effects and prognosis (Reichardt et al., 2021). Disease-modifying therapies (DMTs), which act by suppressing or modulating immune function, are the mainstay of remission-inducing treatment for MS (Hauser and Cree, 2020). For example, Natalizumab, an inhibitor of the adhesion molecule α4β1 integrin, inhibits lymphocyte access to the CNS by blocking adhesion (Yednock et al., 1992), while Fingolimod, Siponimod, and Ozanimodor are sphingosine-1-phosphate [S1P] receptor modulators that sequester lymphocytes in primary lymphoid organs (Calabresi et al., 2014; Kappos et al., 2014; Laroni et al., 2014; Comi et al., 2019; Cohen et al., 2019). Despite the clinical application of these DMTs, effective treatment for MS remains a critical unmet need because current therapies only confer protection against the neurodegenerative manifestations (Hauser and Cree, 2020). Thus, it is urgent to understand the pathogenesis of MS and explore novel preventive and therapeutic strategies.

The current paradigm of MS pathogenesis involves the activation of Th1 and Th17 lymphocytes (Wagner et al., 2020). At the early stage of the disease, peripherally activated T lymphocytes infiltrate the CNS by altering the permeability of the blood-brain barrier (BBB). CD4^+^ T lymphocytes play a crucial role in initiating and maintaining the autoimmune response in MS (Yan et al., 2019), which can differentiate into distinct Th cell populations, including autoreactive Th1/Th17 cells, and secrete the inflammatory cytokines interferon (IFN)-γ and interleukin-17 (IL-17) (Goverman, 2009; Kunkl et al., 2020; Arneth, 2021; Krishnarajah and Becher, 2022). Disruption of BBB integrity is one of the steps in MS pathogenesis and is required for autoreactive T-cell infiltration of the CNS (Sweeney et al., 2019). These auto-active lymphocytes attack the myelin sheath, and alongside inflammation that becomes further amplified and self-sustaining, a spectrum of symptoms occurs, including neuronal damage, neurodegeneration, and demyelination in the brain and spinal cord (Nyirenda et al., 2015; Sospedra and Martin, 2005). Conversely, Th2 and Treg cells play immuno-suppressive roles and can alleviate symptoms and suppress the progression of MS (Wang and Liang, 2022). Moreover, Th1/Th2 and Th17/Treg imbalances are associated with the occurrence and development of various autoimmune diseases, such as MS (Haghmorad et al., 2019; Yu et al., 2019) and rheumatoid arthritis (RA) (Zhang et al., 2023; Yang et al., 2022). Recently, CAR-T cell immunotherapy has been used for the treatment of autoimmune RA by modifying Treg cells to promote their ability to reverse the imbalance of pro- and anti-inflammatory effects. The AMP-activated protein kinase (AMPK) pathway is associated with neurodegeneration and CNS pathology, but the underlying mechanisms involved remain unclear. AMPK dysregulation has been reported in neurodegenerative diseases, such as MS, amyotrophic lateral sclerosis, and Alzheimer’s disease (Muraleedharan and Dasgupta, 2022). Yet, studies on the immunopathological mechanisms and preventive and therapeutic approaches for inflammation-aggravated MS are still scarce.

2′-deoxyinosine (2′-DI) is an endogenous nucleoside formed by the breakdown of deoxyadenosine by the enzyme adenosine deaminase. It has been reported that 2′-DI can inhibit the proliferation of tumor cells, with immunoprotective effects (Yang et al., 2023; Fan et al., 2017). The nucleoside metabolic pathway is involved in the occurrence and development of MS, and several nucleosides and analogs have been approved for MS treatment. For instance, Inosine is beneficial for relapsing-remitting MS and is associated with the blockade of lymphocyte entry into the CNS (Whitmarsh, 2009; Ma et al., 2011; Junqueira et al., 2017); the adenosine receptor antagonist 2-chlorodeoxyadenosine (cladribine) can alleviate symptoms and prevent MS progression (Qi et al., 2023; Mayne et al., 1999). Despite these advances, the potential association between 2′-DI and MS has not yet been investigated. Herein, the present study was designed to explore whether prophylactic 2′-DI intervention could prevent MS progression and elucidate its mechanism of action in this process, with a specific focus on verifying whether 2′-DI exerted preventive immunomodulatory effects through regulating systemic and CNS-localized T helper cell subset balance and limiting inflammatory cell infiltration, and preliminarily exploring the potential association between elevated AMPK phosphorylation and its immunomodulatory function.

## 2 Materials and methods

### 2.1 Sample source

Serum samples were collected from 6 MS patients and 6 age- and gender-matched healthy controls. All enrolled MS patients were diagnosed with relapsing-remitting MS and presented with subacute active relapse phase. MS patients were enrolled based on the following criteria: 1) definite MS diagnosis according to the 2017 McDonald criteria; 2) age ranging from 20 to 50 years; 3) no chronic comorbidities other than MS. All serum samples were freshly collected under fasting conditions on the day of admission and immediately stored at −80 °C to ensure metabolomic stability before detection. All participants provided informed consent form. The study was approved by the Institutional Review Board of Suzhou TCM Hospital Affiliated to Nanjing University of Chinese Medicine.

### 2.2 Peripheral blood lymphocyte isolation

Fresh peripheral blood was collected in a heparin anticoagulant tube and diluted 1:1 in sterile PBS (Gibco, United States). The diluted blood was carefully over an equal volume of Ficoll-Hupaque solution (Thermo Fisher, United States) for density centrifugation. Following centrifugation, the white layer was gently transferred into a new tub and washed with RPMI 1640 medium (Gibco, United States). Subsequently, the cells were maintained in RPMI 1640 complete medium supplemented with 10% fetal bovine serum and 1% streptomycin/penicillin in a humidified incubator at 37 °C under 5% CO_2_ atmosphere for 2 h. This step allows monocytes, which will be adherent to the flask surface, to be separated from the lymphocytes. Finally, the lymphocytes were collected for subsequent experimental analysis. All experiments were performed using primary lymphocytes isolated from at least three independent MS patients as biological replicates to minimize individual biological variability and ensure reliable and reproducible results.

### 2.3 CCK8 and cell apoptosis assays

2′-DI was first pre-dissolved in DMSO to prepare a 1M solution, which was further diluted with RPMI 1640 medium to obtain the desired working concentration for subsequent experiments. The cells were cultured in 96-well plates (100 μL, 1 × 104 cells) and treated with different 2′-DI concentrations (Yuanye, China) for 48 h. To evaluate cell viability, 10 μL of CCK-8 reagent (Vazyme, China) was added, and the optical density of the cells was measured at 450 nm using a microplate reader (BMG, Germany). For cell apoptosis analysis, cells were collected, and resuspended in 100 μL of ice-cold binding buffer by the addition of 5 μL of Annexin V-FITC and 5 μL of PI solution (Vazyme, China). Then, the samples were incubated for 10 min in the dark at room temperature before being subjected to flow cytometry analysis.

### 2.4 T-cell proliferation assay

Lymphocytes were suspended in prewarmed 1 μm CellTraceTM Far Red (CTFR) (Invitrogen, United States)/PBS staining solution at a concentration of 1 × 106 cells/mL and incubated for 15 min in the dark. The reaction was then stopped by adding complete medium, and 1 × 104 CTFR-stained cells were stimulated in 96-well plates with 1 μg/mL OKT-3 and 2 μg/mL CD28 Abs (Sigma, United States) at different concentration of 2′-DI. After 2 days, the CTFR fluorescence of the cells was analyzed using a FACSCanto instrument (BD Biosciences, United States).

### 2.5 Human inflammatory factor assay

The standard curve was constructed according to the manual provided by the Human Inflammatory Factor Kit (BD Sciences, United States). Capture beads, PE detection reagents, and samples (supernatants from the former experiment) were incubated in flow tubes for 3 h at room temperature under light-protected conditions. Subsequently, the binding reaction was terminated by addition of washing buffer and centrifugation. The beads were then resuspended in 300 µL of washing buffer, and PE fluorescence intensity was measured to determine IFN-γ, IL-2, and IL-17 levels using FCAP Array Software.

### 2.6 Animals

Male C57BL/6 mice aged 7 weeks were used in this study, with body weight of 18–20 g. All mice were purchased from Zhejiang Biotechnology Co., Ltd., and housed in the Experimental Animal Center of Soochow University. The mice were fed adaptively for 1 week until the experiment began. The animal experiments were approved by Soochow University’s Animal Care Committee according to the Institutional Animal Care and Use Committee Guidelines.

### 2.7 Induction and administration of experimental autoimmune encephalomyelitis (EAE)

Complete Freund’s adjuvant (CFA) was prepared by mixing *mycobacterium tuberculosis* H-37Ra (MTB) (BD Pharmingen, United States) with incomplete Freund’s adjuvant (Sigma, United States) at a final concentration of 5 mg/mL. For the antigen-adjuvant mixture, 1.5 mg/mL MOG35-55 peptide (BMA, Switzerland) in PBS was emulsified in an equal volume of CFA using syringes attached to a stopcock for 10 min. Then, each mouse received subcutaneous injection of 200 μL emulsion (containing 150 μg MOG35-55 and 500 μg MTB) at four sites. A total of 200 ng of pertussis toxin (Sigma) was intraperitoneally injected into each mouse on days 0 and 2, respectively. After modelling establishment, the mice were randomly divided into the drug administration group administrated with 1 mg/kg of 2′-DI solution (ig. bid) and the vehicle control group treated with the same amount of sterile water. Notably, 2′-DI intragastric administration was initiated on day 1 and lasted until day 23 post-modeling. The drug administration was implemented before the appearance of EAE clinical symptoms, which was defined as preventive intervention.

Throughout the experimental period, the body weight of EAE mice was recorded daily. Clinical EAE scoring was independently assessed by two blinded researchers following standardized grading criteria: 0, no clinical manifestations with intact tail and limb motor function; 1, mild tail hypotonia with normal limb movement; 2, complete tail paralysis and unaffected limbs; 3, tail hypotonia accompanied by hindlimb weakness with intact forelimb function; 4, combined paralysis of the tail, forelimbs and hindlimbs; 5, moribund state or death.

### 2.8 Histopathology

On day 21, the mice were anesthetized with pentobarbitone and perfused with ice-cold PBS. The spinal cord was harvested and embedded in paraffin. Three-micron-thick sections of the spinal cord were stained with H&E to visualize inflammatory infiltration. For inflammation, the following scales were used: 0, none; 1, a few inflammatory cells; 2, organization of perivascular infiltration; and 3, increasing severity of perivascular cuffing with extension into the adjacent tissue.

### 2.9 Mouse cell isolation and culture

The spleen was homogenized, followed by the isolation of spleen lymphocytes using a mouse lymphocyte isolation solution (Thermo Fisher, United States). To isolate lymphocytes from the brain and spinal cord, tissues were sectioned into small fragments, digested with 300 μg/mL collagenase IV (Worthington, United States) and 20 μg/mL DNase I (Roche, Switzerland) for 45 min at 37 °C, and subsequently subjected to density gradient centrifugation using 70% and 30% Percoll-Paque (GE, United States). Cells intended for the analysis of Th1, Th2, and Th17 populations were stimulated with a Cell Stimulation Cocktail (containing protein transport inhibitors) (Invitrogen, United States) for 5 h at 37 °C.

### 2.10 Flow cytometry

To quantify lymphocyte subsets, staining was performed with a viability dye, Ghost-Dye™ Violet 510, along with surface markers CD3 (FITC), CD4 (AF-700), and CD8 (PerCP-5.5) at 4 °C for 30 min in the dark. Following surface staining, cells were fixed and permeabilized using a Cytofix/Cytoperm Plus kit (BD Biosciences). Subsequently, intracellular staining was conducted with anti-Foxp3 Abs (APC), anti-IFN-γ Abs (PE-Cy7), anti-IL-4 Abs (BV605), and anti-IL-17A Abs (PE). All antibodies were sourced from BD Biosciences. The BD FACSVerse™ system and FlowJo™ V10 software were used for data analysis. The representative gating strategy for all flow cytometric analyses is provided in Supplementary Figure S1.

### 2.11 RNA extraction, cDNA synthesis, and quantitative real-time PCR

Total RNA was isolated from lymphocytes by TRIzol reagents (15596018CN, Invitrogen), and cDNA was synthesized using 5 × All-In-One RT MasterMix (G492, ABM). Quantitative PCR was performed using SYBR Green reagents (820A, Takara). RNA expression was calculated using GAPDH expression as a standard by the ΔCt method, and the results were expressed as the fold change and were normalized to the control. The sequences of primers used are listed in Table 1.

**TABLE 1:** The primer sequences used for qRT-PCR.

Gene names	Species	Forward primer (5ʹ-3ʹ)	Reverse primer (5ʹ-3ʹ)
GAPDH	Human	GTC​TCC​TCT​GAC​TTC​AAC​AGC​G	ACC​ACC​CTG​TTG​CTG​TAG​CCA​A
FOXP3	Human	GGC​ACA​ATG​TCT​CCT​CCA​GAG​A	CAG​ATG​AAG​CCT​TGG​TCA​GTG​C
IFN-γ	Human	GCT​TTT​CAG​CTC​TGC​ATC​GT	CCG​CTA​CAT​CTG​AAT​GAC​CTG
GAPDH	Mouse	GGT​TGT​CTC​CTG​CGA​CTT​CA	TGG​TCC​AGG​GTT​TCT​TAC​TCC
FOXP3	Mouse	CCT​GGT​TGT​GAG​AAG​GTC​TTC​G	TGC​TCC​AGA​GAC​TGC​ACC​ACT​T
IFN-γ	Mouse	CTG​GAG​GAA​CTG​GCA​AAA​GGA​TGG	GACGCITATGITGITGCTGATGGC
IL-4	Mouse	ACG​GAG​ATG​GAT​GTG​CCA​AAC	AGC​ACC​TTG​GAA​GCC​CTA​CAG​A
IL-17	Mouse	AAA​GCT​CAG​CGT​GTC​CAA​AC	TGG​AAC​GGT​TGA​GGT​AGT​CTG
CXCR3	Mouse	GCT​GTA​GCC​GAT​GTT​CTG​CTG​GTG	TGC​ACT​ATG​CTC​AGA​TAT​CTG​TC

### 2.12 RNA sequencing

Total RNA was extracted from lymphocytes of healthy individuals, followed by library preparation according to Illumina standard instructions (VAHTS Universal V6 RNA-seq Library Prep Kit for Illumina®). An Agilent 4200 bioanalyzer was used to evaluate the concentration and size distribution of the cDNA library before sequencing with an Illumina NovaSeq 6000 instrument. The raw reads were filtered by Seqtk before they were mapped to the genome using HISAT2 (version 2.0.4) (Kim et al., 2015). The gene fragments were counted using Stringtie (v1.3.3b) followed by trimmed mean of M values (TMM) normalization (Mortazavi et al., 2008; Pertea et al., 2015; Pertea et al., 2016; Robinson and Oshlack, 2010). Significantly differentially expressed genes (DEGs) were identified as those with a false discovery rate (FDR) value above the threshold (Q < 0.05) and a fold change >2 using edgeR software (Benjamini and Hochberg, 1995; Benjamini and Yekutieli, 2001; Robinson et al., 2010).

### 2.13 Serum metabolomics analysis

Serum samples were collected from MS patients and healthy controls. Water, methanol, acetonitrile, and formic acid were purchased from Thermo Fisher Scientific (Thermo Fisher Scientific, Waltham, MA, United States). L-2-chlorophenylalanine was obtained from Shanghai Heng Chuang Biotechnology Co., Ltd. (Shanghai, China). The samples were analyzed on LC-MS/MS platforms by Lumingbio. For LC-MS/MS, samples were reconstituted in acidic or basic LC-compatible solvents containing >11 injection standards and run on a Waters ACQUITY UPLC and Thermo-Finnigan LTQ mass spectrometer with a linear ion-trap front-end and a Fourier transform ion cyclotron resonance mass spectrometer back-end. Chemical entities were identified by comparison to the metabolomic library entries of purified standards. Following log transformation and imputation with minimum observed values for each compound, the data were analyzed using one-way analysis of variance (ANOVA) to test for group effects. P and q values were calculated based on two-way ANOVA contrasts. Principal component analysis was used to visualize variance distributions.

### 2.14 Western blot analysis

Lysis buffer (Beyotime, China) and the protease inhibitor phenylmethyl sulfonyl fluoride (Beyotime, China) were added to Eppendorf tubes containing spleen lymphocytes, brains, and spinal cords. Then, the homogenates were subjected to ultrasonication at 4 °C for half an hour. After centrifugation, the supernatant was collected, and the protein concentration was determined with a BCA protein assay kit (Beyotime, China). Equal amounts of protein were resolved by electrophoresis on 12.5% gradient polyacrylamide gels and transferred to nitrocellulose membranes. Nitrocellulose membranes were blocked with 5% skim milk for 1 h at room temperature and incubated with primary antibodies overnight at 4 °C. After washing, the membranes were incubated with horseradish peroxidase-conjugated secondary antibodies (Proteintech, China). Chemiluminescent bands were detected with an ECL Western Blotting Substrate Kit (Proteintech, China) in a blot scanner (Bio-Rad, China). Densitometric analysis was performed for quantification using ImageJ software. In this study, all primary antibodies were purchased from CST (United States).

### 2.15 Statistical analysis

The presented results are representative of experiments repeated at least three times. Data are expressed as the means ± standard deviation (SD), and normality is assessed via the Shapiro-Wilk test. For two-group comparisons, two-tailed paired/unpaired *t*-tests or Mann-Whitney U tests are selected according to data distribution. Comparisons across three or more groups are conducted using one-way or two-way ANOVA followed by Dunnett’s *post hoc* test for multiple comparison correction, with adjusted *p* values reported. Statistical significance was defined as *p* > 0.05 (not significant, NS), *p* < 0.05 (*), *p* < 0.01 (**), *p* < 0.001 (**8), and *p* < 0.0001 (****). All statistical analyses are performed with GraphPad Prism 6 (GraphPad Software Inc., United States) and R 4.2.2 (RStudio/Posit Inc., United States).

## 3 Results

### 3.1 MS patients display a reduced level of 2′-DI

Based on LC-MS/MS, it was observed that the differentially abundant metabolites were significantly enriched in the nucleoside metabolic pathway, especially in the 2′-DI, which was decreased significantly in MS patients compared with healthy controls (Figures 1A–C). Deoxyadenosine, a nucleoside of this pathway, was also markedly reduced in MS patients (Figure 1C). Moreover, the levels of two nucleoside metabolic components adenosine and inosine, which are involved in MS treatment and pathogenesis, were found to decrease slightly in MS patients (Figure 1C). These results indicated that decreased 2′-DI levels might be involved in the progression of MS.

**FIGURE 1:**
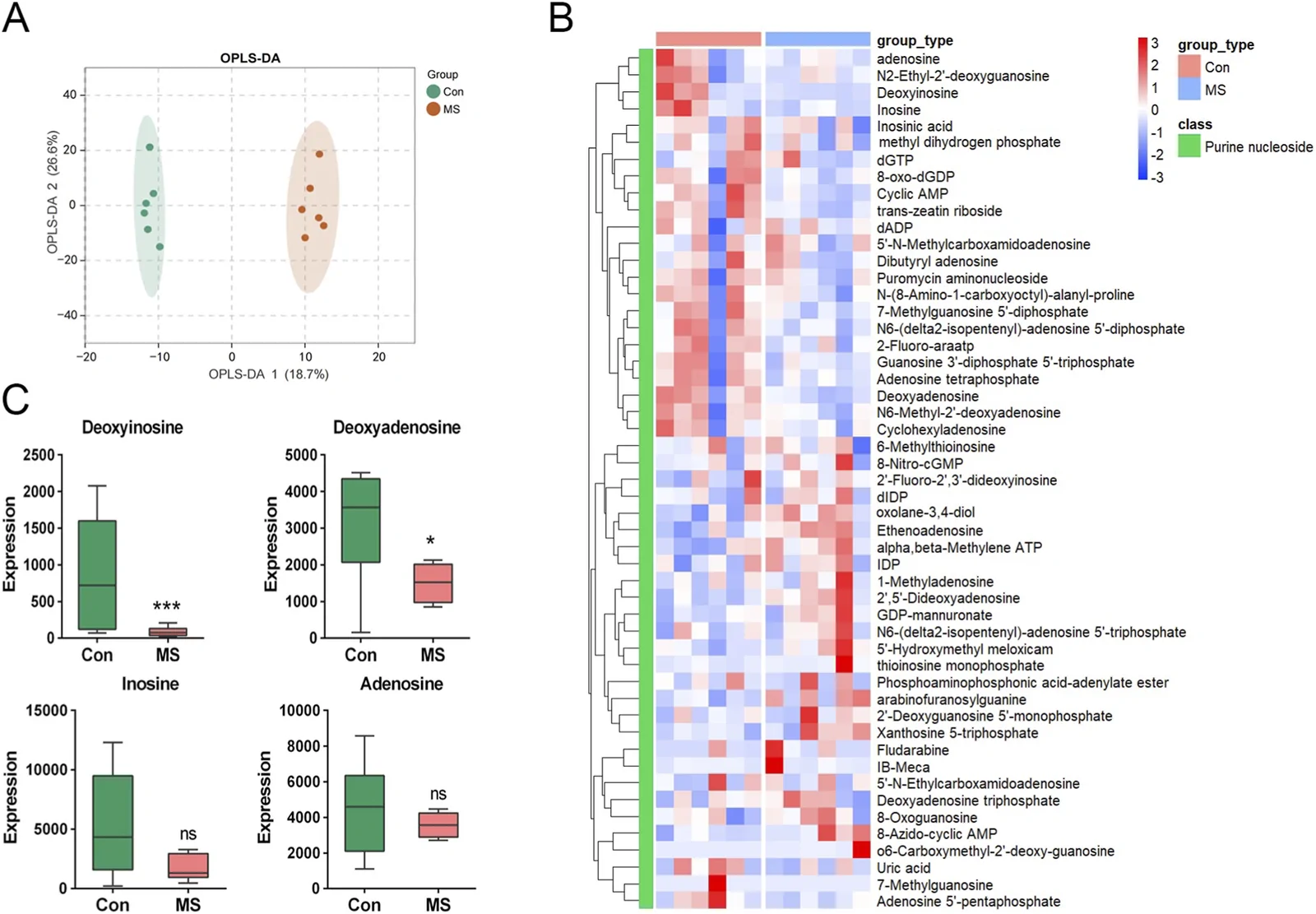
Metabolomic patterns of MS patients and healthy controls (n = 6). **(A)** The OPLS-DA model for discriminating the MS and Con groups; **(B)** Heatmap of the changes in the purine nucleoside pathway between the MS and Con groups (OPLS-DA VIP >1.77); **(C)** The expression levels of deoxyinosine, deoxyadenosine, inosine and adenosine in MS patients and healthy controls. *p < 0.05, **p < 0.01, ***p < 0.001.

### 3.2 2′-DI prophylactic intervention effectively prevents MS progression

Considering decreased 2′-DI levels in MS patients, we hypothesized that supplementation with 2′-DI could prevent MS progression. To verify this, EAE mice were divided into the 2′-DI group and the vehicle control group. Compared with the vehicle control, the EAE mice receiving prophylactic 2′-DI had stable body weights, reduced disease severity scores, and decreased disease incidence (Figures 2A–D). H&E results showed that the tissue architecture in the vehicle group was markedly disorganized. Lesion areas exhibited loose tissue arrangement, vacuolar degeneration and edematous changes, accompanied by extensive inflammatory cell infiltration. In contrast to the vehicle group, the spinal cord tissue in the 2′-DI group displayed well-preserved morphological features with relatively compact tissue structure. The degrees of vacuolar degeneration and inflammatory infiltration were alleviated, and tissue destruction within the lesion regions was milder. LFB-positive blue staining was markedly attenuated and unevenly distributed in the vehicle group. The white matter exhibited loose tissue architecture, prominent vacuolation and disorganized myelin arrangement. Numerous punctate and clumpy purple counterstaining signals were visible under a 40× objective lens; their accumulation indicates augmented local cellular reactions and exacerbated inflammatory infiltration secondary to injury. Compared with the vehicle group, the 2′-DI group showed markedly intensified and more continuous LFB staining, accompanied by relatively compact tissue architecture, alleviated vacuolation, and better-preserved myelin structures (Figure 2E). These results suggest that prophylactic administration of 2′-DI may inhibit inflammatory cell infiltration and preserve myelin structures in the spinal cords of EAE mice. Subsequently, we further investigated lymphocyte levels in the central and peripheral immune organs of EAE mice. It was observed that the EAE mice treated with 2′-DI showed lower lymphocyte counts in the spinal cord, brain and peripheral blood, but higher counts in the spleen (Figures 2F–I). Taken together, prophylactic 2′-DI intervention significantly attenuated clinical symptoms and inflammatory infiltration in EAE mice, verifying its preventive efficacy against MS.

**FIGURE 2:**
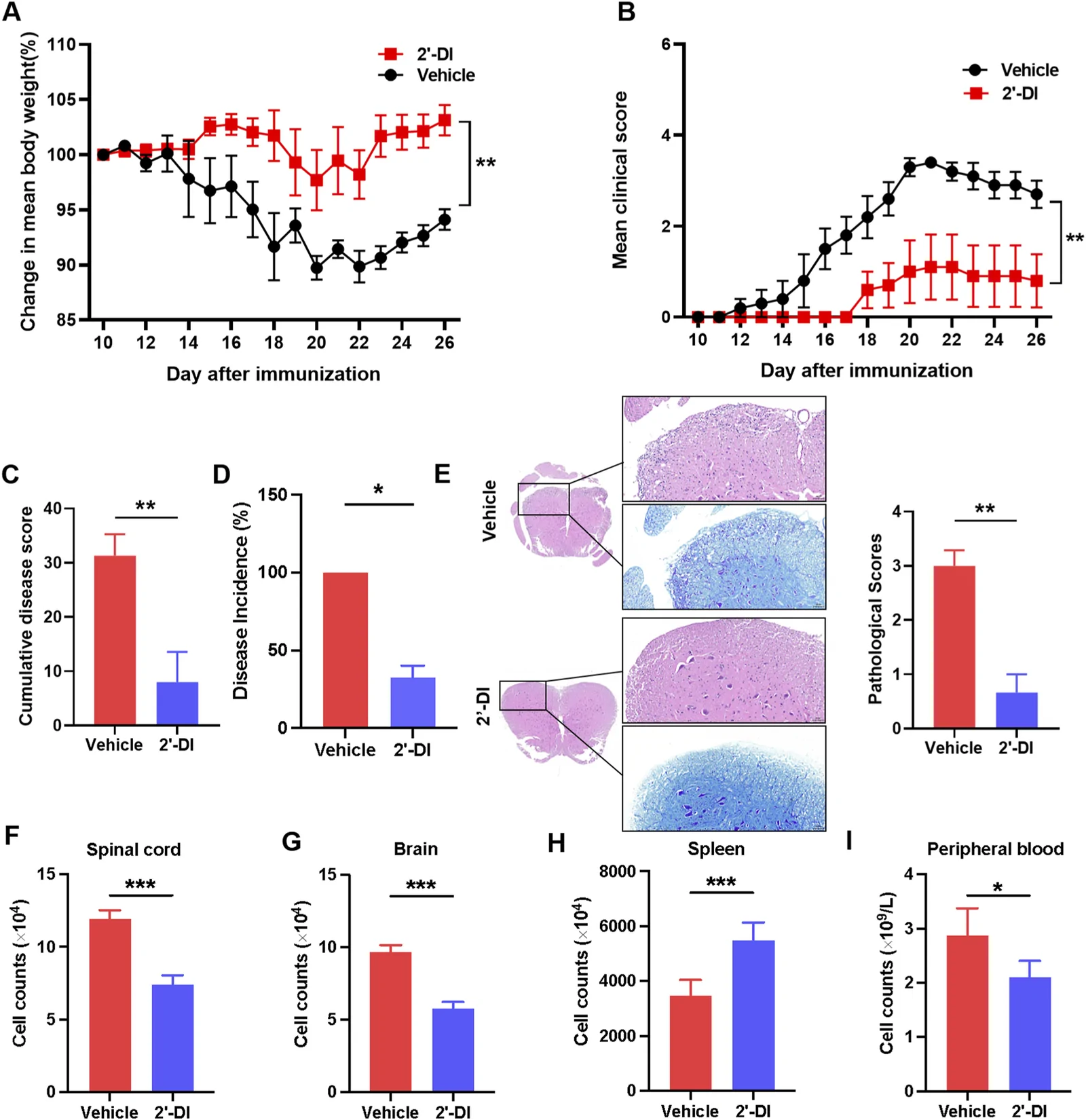
Administration of 2′-DI inhibits disease progression in the EAE model (n = 5). **(A)** The mean body weight following treatment with 2′-DI and the vehicle control. **(B)** The mean EAE severity scores (ranging from symptom observable disease to moribund/dead) and cumulative EAE severity score **(C)** following treatment with 2′-DI and vehicle. **(D)** Disease incidence (%) following treatment with 2′-DI and the vehicle control. **(E)** Hematoxylin & eosin (H&E) and Luxol Fast Blue (LFB) staining of the spinal cord at the acute phase of EAE following treatment with 2′-DI and vehicle. Scale bars (4× magnification) = 200 μm, scale bars (40× magnification) = 50 μm. **(F)** Lymphocyte counts in the spinal cord, brain **(G)**, spleen **(H)**, and peripheral blood **(I)** of EAE mice after treatment with 2′-DI and vehicle. The presented results are representative of experiments repeated at least three times. The data are presented as the mean ± SD. *p < 0.05, **p < 0.01, ***p < 0.001.

### 3.3 2′-DI treatment shifts the transcriptomic profiles of MS

To determine the underlying preventive function of 2′-DI in MS pathogenesis, lymphocytes from MS patients were cultured *in vitro*. Transcriptomic profiling analysis was conducted after lymphocytes were treated with 0, 0.5, or 1 mM 2′-DI and OKT3 as the control group. Compared with the control group, DEG analysis revealed increased expression of 24 genes and decreased expression of 60 genes in the 0.5 mM DI group (Supplementary Figure S2). Significantly, 83 genes were upregulated, and 566 genes were downregulated in the 1 mM DI group (Figure 3A), suggesting that 2′-DI might affect gene expression in a dose-dependent manner. Gene Ontology analysis revealed that the genes related to chemokine receptor binding, chemokine activity, and T-cell chemotaxis were significantly enriched (Figure 3B). Moreover, we also found that 15 proinflammatory cytokines and receptors, 16 chemokines, and 7 chemokine receptors were downregulated with the increasing 2′-DI dose (Figure 3C). The Kyoto Encyclopedia of Genes and Genomes (KEGG) analysis showed that the immune system, signal transduction, cell growth, and death process terms were markedly enriched (Supplementary Figure S2). Among the top 30 KEGG pathways, the IL-17 signaling pathway, p53 signaling pathway, cytokine, and cytokine receptor interaction, chemokine signaling pathway, and cell cycle were significantly enriched (Figure 3D), indicating that 2′-DI might play a preventive immunoregulatory role in MS development.

**FIGURE 3:**
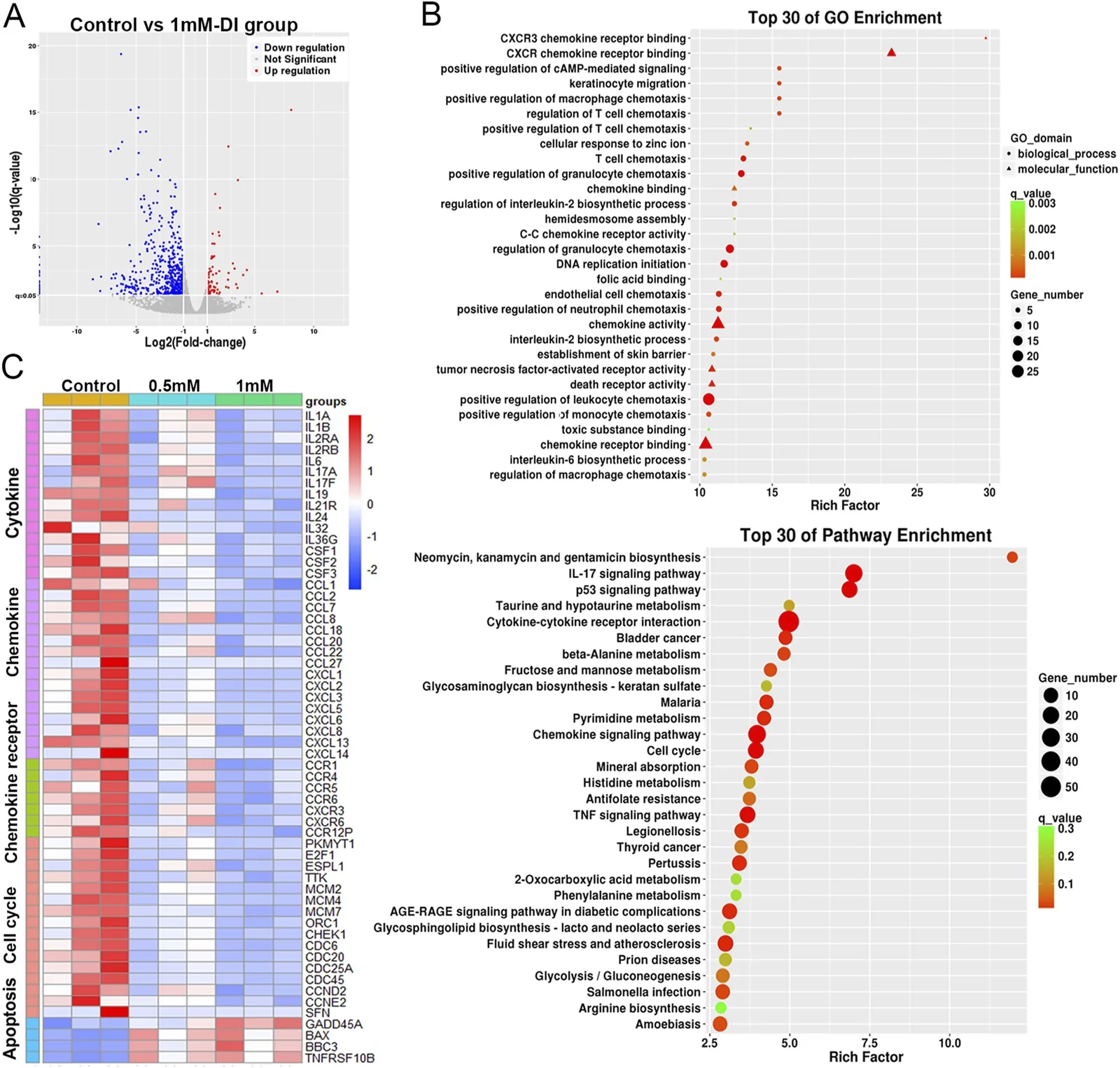
2′-DI administration shifts the transcriptomic profiles of MS (n = 3). **(A)** Volcano plot of the DEGs between the control and 1 mM-DI groups. **(B)** GO and KEGG enrichment analyses of the control and 1 mM-DI groups. **(C)** Heatmap analysis of the DEGs related to inflammatory cell activation, migration, and infiltration with increasing 2′-DI doses. Control: peripheral blood supplemented with 0, 0.5 (0.5 mM-DI group), or 1 mM 2′-DI (1 mM-DI group).

### 3.4 2′-DI inhibits lymphocyte proliferation and promotes cell apoptosis *in vitro*

After treatment with 2′-DI *in vitro*, the lymphocyte proliferation rates decreased significantly, while the cell apoptosis rate increased in a dose-dependent manner (Figures 4A,B; Supplementary Figures S3A,B). Meanwhile, the levels of IFN-γ, IL-17 and IL-2 were also decreased in a dose-dependent manner (Figure 4C). To further validate these findings, qRT-PCR analysis was performed, and the results demonstrated that 2′-DI inhibited the expression of IFN-γ (Supplementary Figure S3C). Notably, the expression of the key regulatory factor Foxp3 in Treg cells, which suppresses immune activation, was increased significantly at the mRNA level after treatment with 0.5 mM 2′-DI (Supplementary Figure S3D). These results indicate that 2′-DI can inhibit the proliferation of lymphocytes and the secretion of proinflammatory factors *in vitro*, supporting its *in vivo* preventive immunomodulatory effects on MS.

**FIGURE 4:**
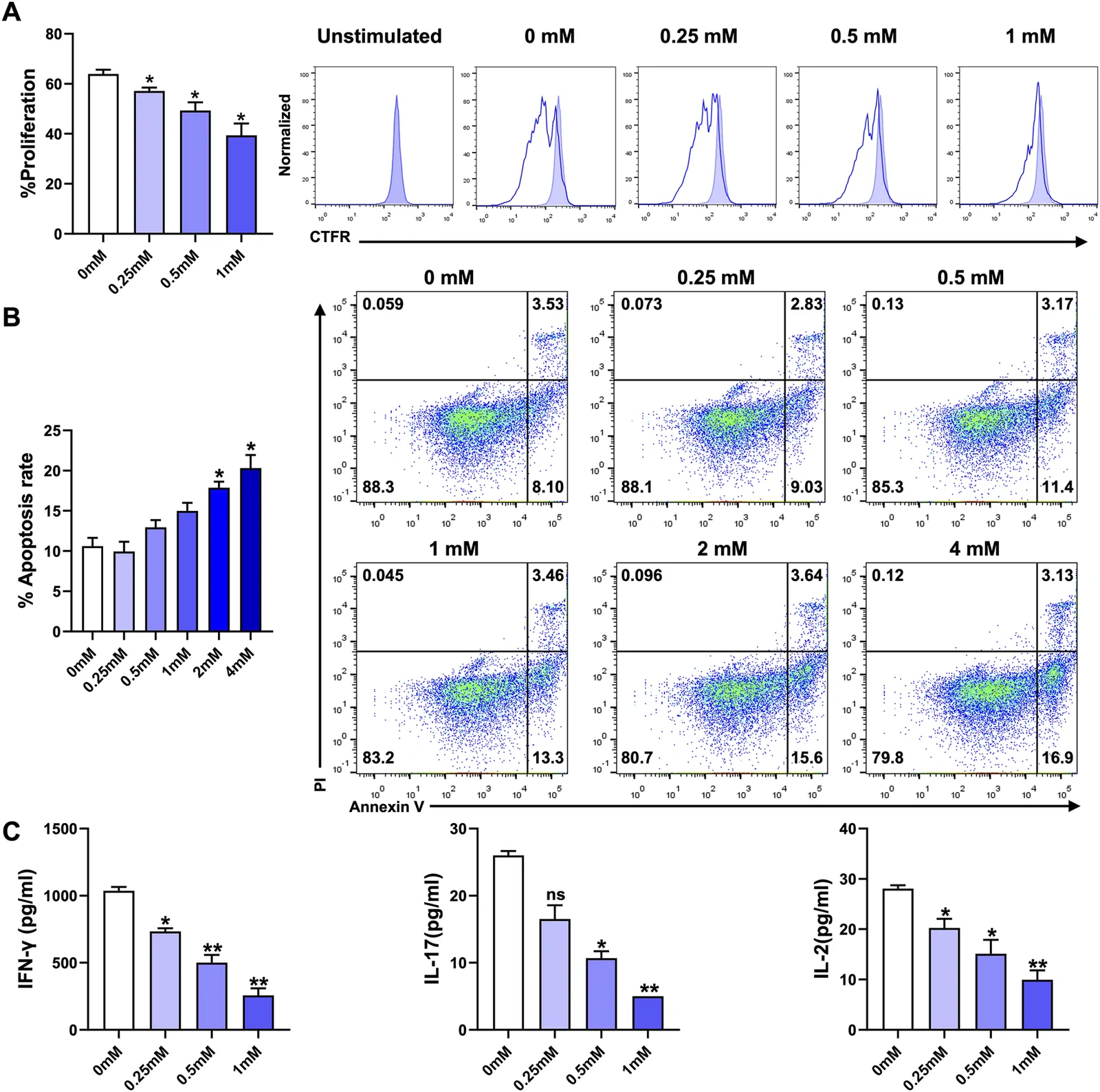
Administration of 2′-DI inhibits cell proliferation and promotes lymphocyte apoptosis. **(A)** CTFR analysis of lymphocyte proliferation after treatment with 0, 0.25, 0.5, or 1 mM 2′-DI (n = 5). **(B)** Administration of 2′-DI promotes lymphocyte apoptosis (n = 3). **(C)** Administration of 2′-DI inhibits the secretion of proinflammatory cytokines (n = 4). The presented results are representative of experiments repeated at least three times. The data are presented as the mean ± SD. ns = no significance, *p < 0.05, **p < 0.01.

### 3.5 2′-DI promotes the balance of Th1/Th2 and Th17/Treg cells in the spinal cord

The spinal cord is one of the main sites of MS, and the disease begins when activated T lymphocytes infiltrate the CNS by altering the permeability of the BBB. First, we investigated the preventive immunoregulatory function of 2′-DI in the spinal cord. T-cell subset analysis demonstrated that the percentages of CD3^+^ T cells, CD4^+^ T cells, CD4^+^ Ki67+ cells and CD8^+^ IFN-γ+ cells in the 2′-DI group were significantly lower than those in the vehicle group (Supplementary Figures S4A,B).

An imbalance of Th1/Th2 and Th17/Treg cells plays a dominant role in the pathogenesis of MS, resulting in neuronal damage in the brain and spinal cord (Haghmorad et al., 2022; Liu et al., 2022). We then analyzed the percentages of Th1 (CD4^+^ IFN-γ+), Th2 (CD4^+^ IL-4+), Th17 (CD4^+^ IL-17+), and Treg (CD4^+^ Foxp3+) cells differentiated from CD4^+^ T cells. Compared with those in the control group, the proportions of Th1 and Th17 cells were decreased markedly in the 2′-DI group, while those of Th2 and Treg cells increased (Figures 5A,B). Additionally, the expression of IFN-γ, IL-17, and CXCR3 was decreased markedly, whereas that of Foxp3 and IL-4 was increased in the 2′-DI group compared with the vehicle group (Figure 5C). Thus, 2′-DI regulates the differentiation of CD4^+^ T cells and decreases the inflammatory response of the spinal cord by promoting the balance of Th1/Th2 and Th17/Treg cells, thereby exerting preventive effects on MS neuroinflammation.

**FIGURE 5:**
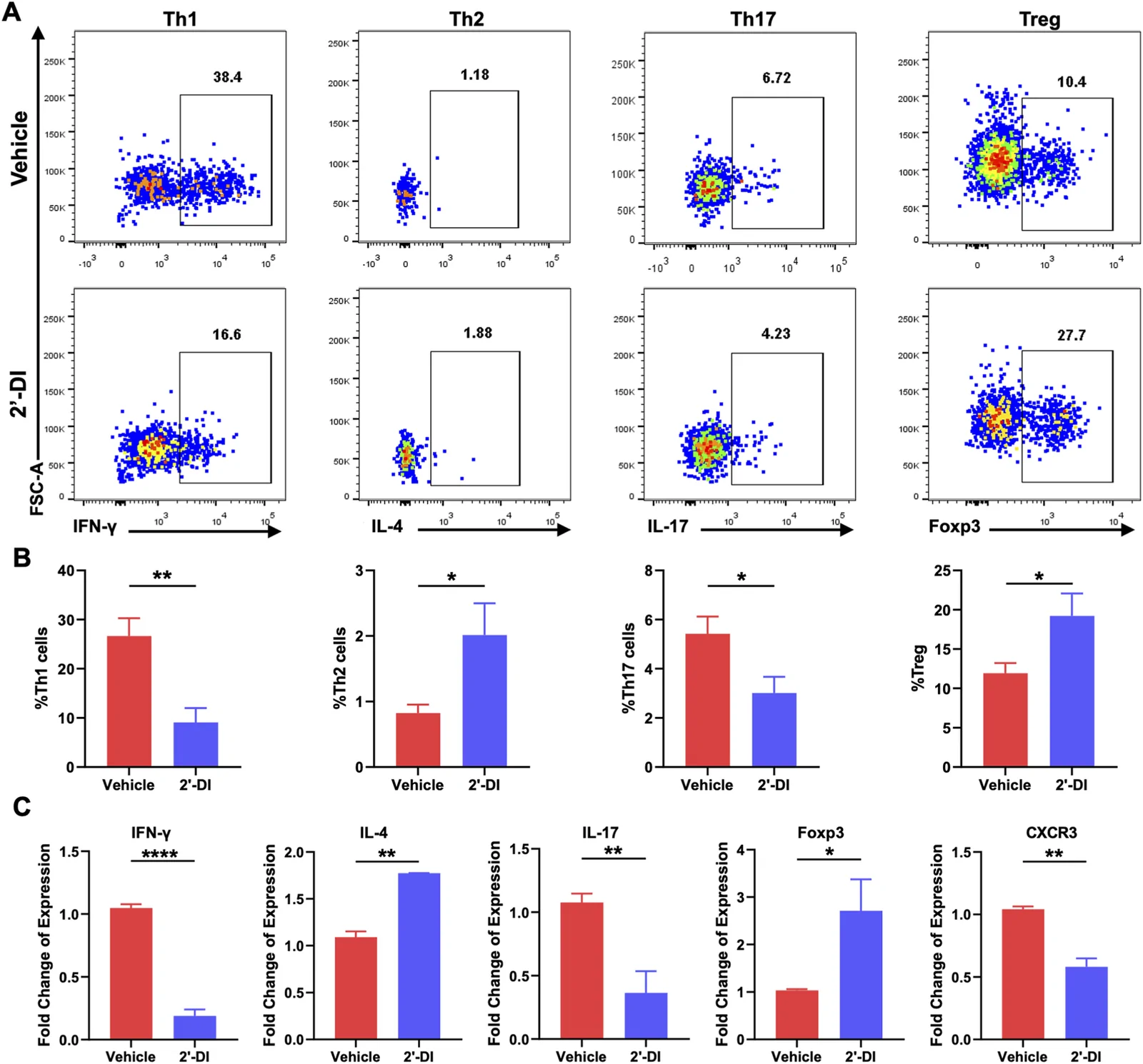
Administration of 2′-DI promotes the balance of Th1/Th2 and Th17/Treg cells in the spinal cord of EAE mice. **(A)** Representative density plot and bar graph **(B)** for flow cytometry analysis of Th1/Th2/Th17/Treg cell populations differentiated by CD4^+^ T cells in the spinal cord of EAE mice treated with vehicle (VE) or 2′-DI (DI) (n = 5). **(C)** qRT-PCR analysis of representative pro- and anti-inflammatory cytokine/transcription factor/chemokine receptor expression in the spinal cord of EAE mice treated with vehicle or 2′-DI (n = 4). The data are presented as the mean ± SD. ns = no significance, *p < 0.05, **p < 0.01.

Notably, by quantifying the absolute counts of Th1, Th2, Th17, and Treg cells within the spinal cord, we observed that the absolute counts of Th1 and Th17 cells were markedly decreased after 2′-DI intervention, while Th2 and Treg absolute numbers showed no significant variations. This phenomenon may be biologically predictable, as 2′-DI intervention could reduce the infiltration of T cells into the CNS.

### 3.6 2′-DI facilitates the balance of Th1/Th2 and Th17/Treg cells in the brain

Similar to the findings in the spinal cord, 2′-DI inhibited the proliferation and activation of T cells, as shown by the significant decreases in CD3^+^ T cells, CD4^+^ T cells, and CD8^+^ IFN-γ+ cells, except for CD4^+^ Ki67+ cells (Supplementary Figure S5). By analyzing the CD4^+^ T-cell subtypes, we found that 2′-DI administration could decrease the number of Th1 and Th17 cells but increase the number of Th2 and Treg cells (Figures 6A,B). Furthermore, the expression of IFN-γ, IL-17, and CXCR3 were decreased significantly, but that of IL-4 and Foxp3 was increased in the 2′-DI group (Figure 6C). Overall, 2′-DI intervention could decrease the inflammatory response in the brain by relieving the imbalance in Th1/Th2 and Th17/Treg cells.

**FIGURE 6:**
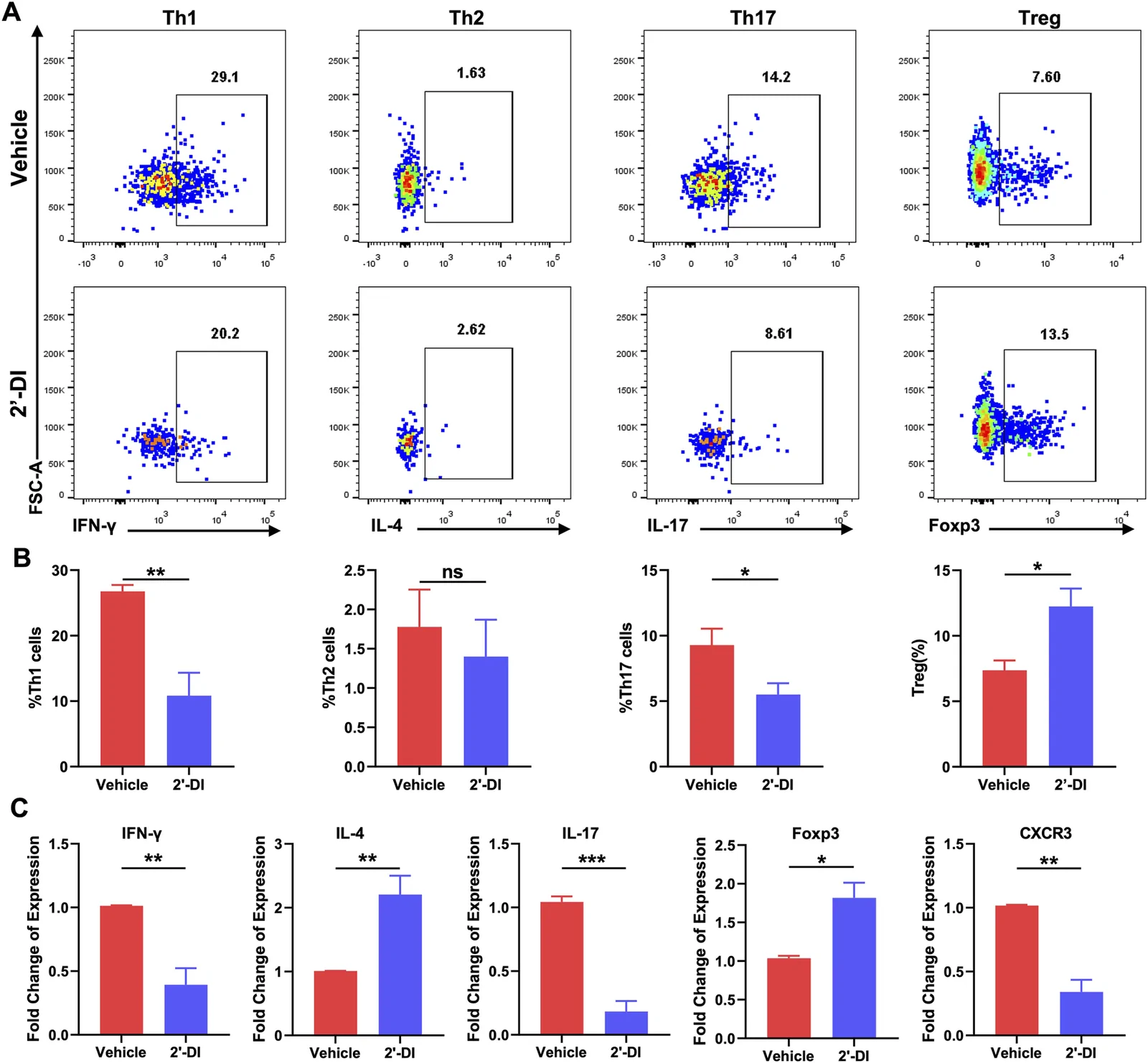
Administration of 2′-DI promotes Th1/Th2 and Th17/Treg balance in the brain of EAE mice. **(A)** Representative density plot and bar graph **(B)** for flow cytometry analysis of Th1/Th2/Th17/Treg cell populations differentiated by CD4^+^ T cells in the brain of EAE mice treated with vehicle or 2′-DI (n = 5). **(C)** qRT-PCR analysis of representative pro- and anti-inflammatory cytokine/transcription factor/chemokine receptor expression in the brain of EAE mice treated with vehicle or 2′-DI (n = 4). The presented results are representative of experiments repeated at least three times. The data are presented as the mean ± SD. ns = no significance, *p < 0.05, **p < 0.01, ***p < 0.001.

### 3.7 2′-DI enhances the balance of Th1/Th2 and Th17/Treg cells in both the spleen and peripheral blood

To investigate whether 2′-DI could inhibit the activation of peripheral T lymphocytes to reduce immune infiltration in the CNS, we analyzed the T-cell subsets in the spleen and peripheral blood. As shown in Supplementary Figure S6, CD3^+^ T cells were decreased in both the spleen and peripheral blood after 2′-DI administration. Notably, no significant difference in splenic CD4^+^ T cell count was detected between the 2′-DI and vehicle groups. However, the number of peripheral blood CD4^+^ T cells was significantly decreased following 2′-DI treatment. Moreover, the number of Treg cells were decreased in the peripheral blood after 2′-DI administration, which was observed alongside improved systemic immune homeostasis.

Furthermore, 2′-DI could reduce the percentage of Th1 and Th17 cells and increase the percentage of Treg cells in the spleen between the 2′-DI and vehicle groups, but without significant differences in the percentage of Th2 cells (Figures 7A,B). At the transcriptional level, 2′-DI administration downregulated the expression of IFN-γ, IL-17, and CXCR3, while upregulating that of Foxp3 and IL-4 relative to the control group (Figure 7C). These findings indicate that 2′-DI treatment remodels peripheral immune cell subsets and may reduce immune cell infiltration into the CNS via indirect immunomodulatory effects, thereby preventing the progression of MS.

**FIGURE 7:**
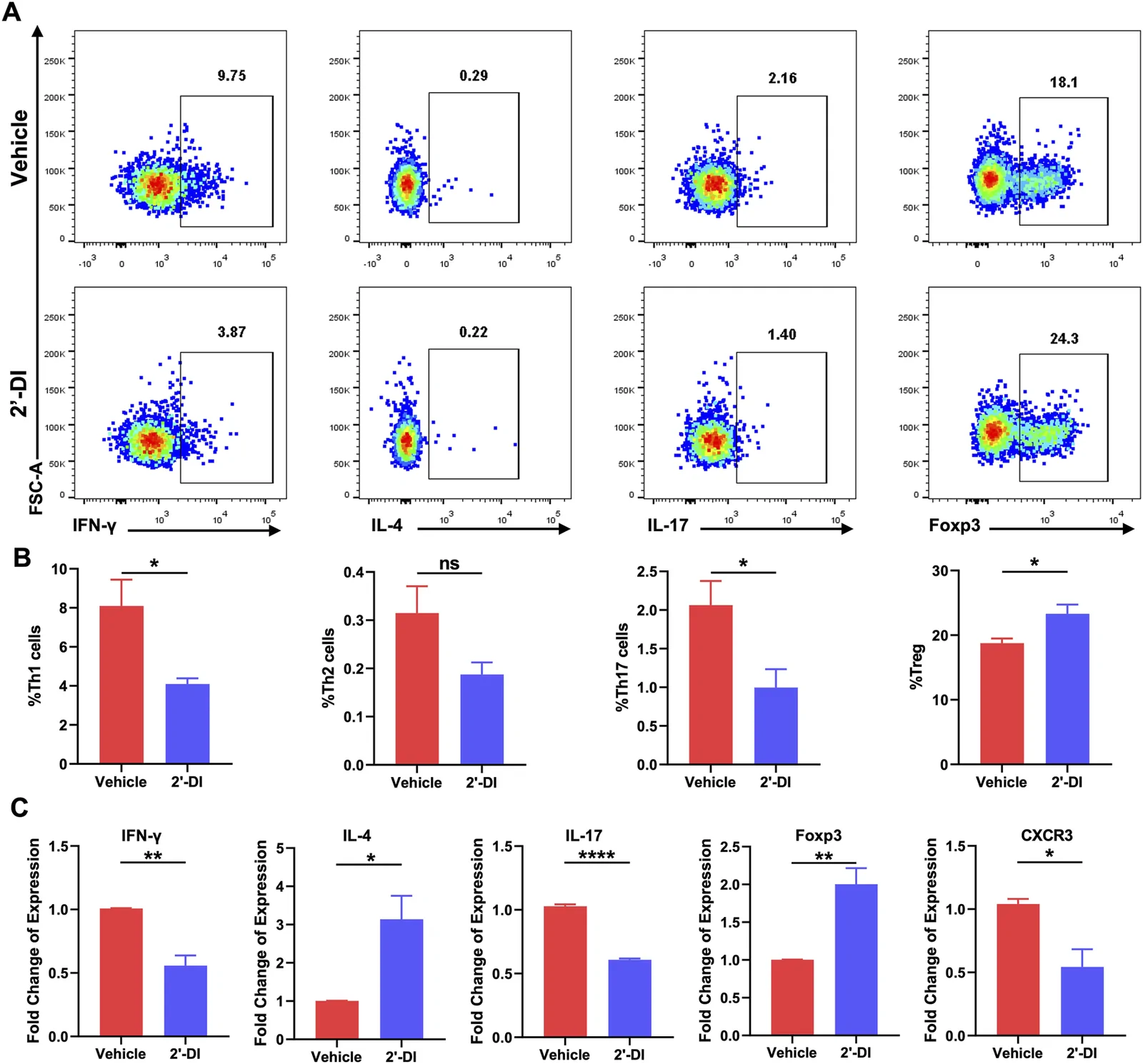
Administration of 2′-DI promotes Th1/Th2 and Th17/Treg balance in the spleen of EAE mice. **(A)** Representative density plot and graph **(B)** for flow cytometry analysis of Th1/Th2/Th17/Treg cell populations differentiated by CD4^+^ T cells in the spleen of EAE mice treated with vehicle or 2′-DI (n = 5). **(C)** qRT-PCR analysis of representative pro- and anti-inflammatory cytokine/transcription factor/chemokine receptor expression in the spleen of EAE mice treated with vehicle or 2′-DI (n = 4). The data are presented as the mean ± SD. ns = no significance, *p < 0.05, **p < 0.01, ***p < 0.001.

### 3.8 Elevated AMPK phosphorylation is potentially associated with 2′-DI-mediated modulation of pro-inflammatory and anti-inflammatory immune balance

The AMPK and NF-κB signaling pathways are crucial modulators of MS immunopathogenesis. Previous studies have demonstrated that activated AMPK signaling promotes Treg expansion and exerts neuroprotective effects in MS, while hyperactivated NF-κB p65 triggers robust pro-inflammatory cytokine secretion and exacerbates neuroinflammatory damage and disease progression (Kong et al., 2016; Mc Guire et al., 2013; Zhou et al., 2020). As a central transcription factor driving Th1 and Th17 cell differentiation, NF-κB p65 directly binds the promoter regions of IFN-γ, T-bet, and RORγt, and integrates upstream signals including TCR signaling and the IL-12/IL-6/IL-23 inflammatory cascade (Ruan et al., 2011). Inhibition of NF-κB activity markedly suppresses Th1 and Th17 immune reactions; conversely, excessive NF-κB activation antagonizes the expression of Foxp3, a master regulator of Treg development and function (Xie et al., 2019).

To further explore the molecular mechanism of 2′-DI in regulating pro-inflammatory and anti-inflammatory immune homeostasis and mediating its preventive effects on MS, we detected the protein expression and phosphorylation levels of key molecules in the AMPK and NF-κB pathways via Western blotting. The results showed that 2′-DI treatment did not affect the total protein expression of AMPK, but significantly increased AMPK phosphorylation. By contrast, neither the total expression nor phosphorylation level of NF-κB p65 exhibited obvious alterations following 2′-DI intervention (Figure 8). These data indicate a potential association between 2′-DI-induced elevation of AMPK phosphorylation and the restoration of T cell subset immune balance. Taken together, 2′-DI-mediated immunomodulatory effects on pro-inflammatory and anti-inflammatory immune homeostasis are potentially linked to elevated AMPK phosphorylation status, which underpins its preventive effects against MS.

**FIGURE 8:**
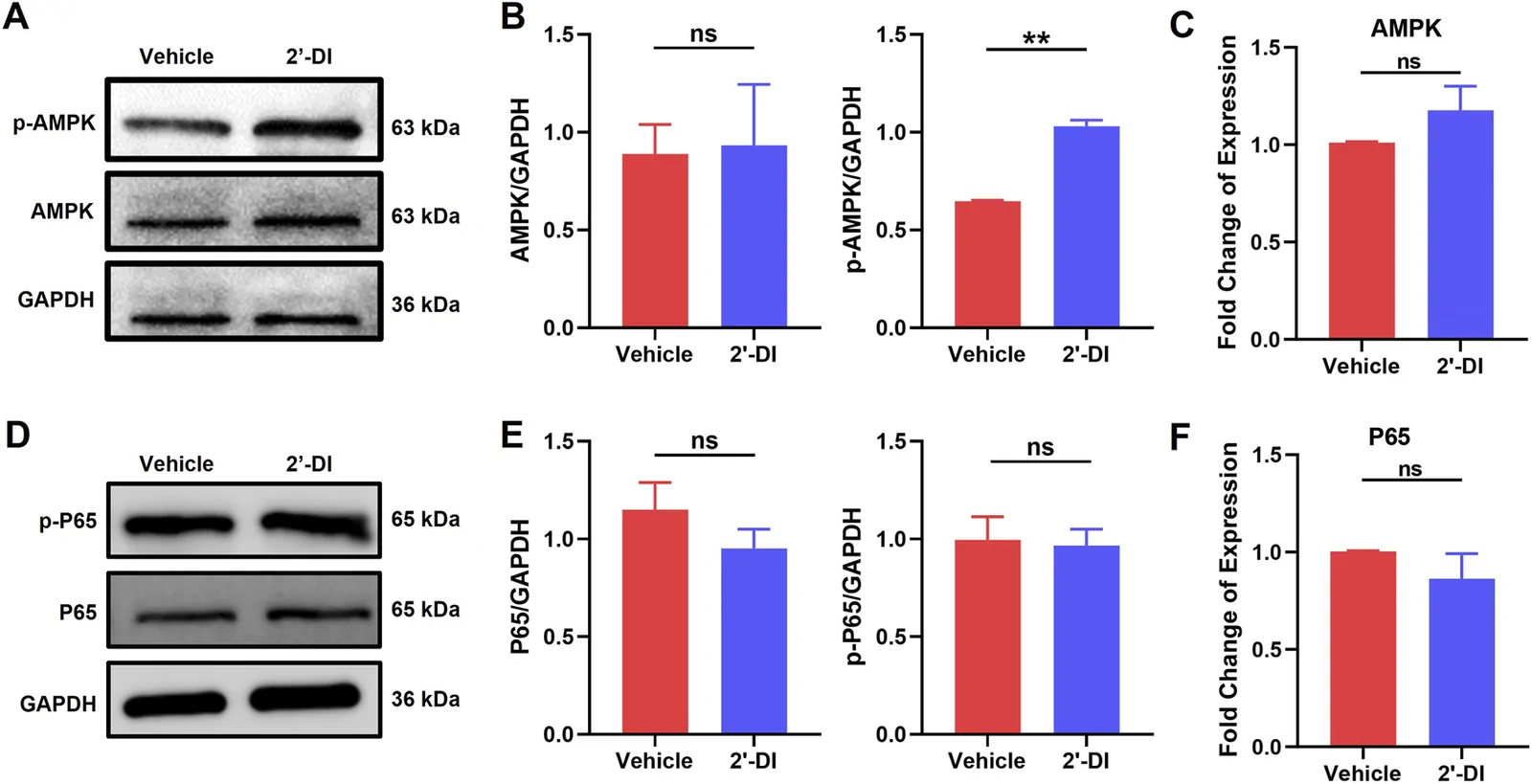
Expression of the upstream regulators AMPK and P65 in EAE mice after treatment with 2’-DI or vehicle (n = 3). **(A–C)** Protein expression of AMPK and p-AMPK, and AMPK mRNA expression determined by qRT-PCR in the spleen of EAE mice. **(D–F)** Protein expression of P65 and p-P65, and P65 mRNA expression determined by qRT-PCR in the spleen of EAE mice. The presented results are representative of experiments repeated at least three times. The data are presented as the mean ± SD. ns = no significance *p < 0.05, **p < 0.01, ***p < 0.001.

## 4 Discussion

In the present study, we identified a significant reduction in serum 2′-DI levels in patients with MS, an autoimmune neuroinflammatory disorder characterized by chronic demyelination and neurodegeneration. This decrease in 2′-DI was closely associated with exacerbated inflammatory responses driven by proinflammatory Th1 and Th17 cells, alongside impaired function of anti-inflammatory Treg and Th2 cells. Collectively, the reduction of 2′-DI in MS patients, accompanied by consequent immune dysregulation, may serve as a potential contributing factor that that potentially facilitates neurodegenerative processes during MS progression. Furthermore, our *in vitro* and *in vivo* findings consistently demonstrated that exogenous 2′-DI supplementation could effectively prevent MS progression via prophylactic intervention, which was mediated by 2′-DI-induced polarization toward immunosuppressive Treg and Th2 cells, coupled with the suppression of pro-inflammatory Th1 and Th17 cell activation. Importantly, the immunomodulatory effects of 2′-DI were found to be correlated with elevated AMPK phosphorylation status. Notably, the preventive benefits of 2′-DI were exclusively derived from the inhibition of autoimmune inflammatory cascades, and there was no evidence indicating that 2′-DI directly repaired demyelinating lesions or alleviated axonal injury in EAE mice. Mechanistically, 2′-DI appears to mitigate MS-related pathological phenotypes primarily through two core immunoregulatory actions: blocking the infiltration of autoreactive lymphocytes into the CNS and restoring the disrupted Th1/Th2 and Th17/Treg immune balance in both peripheral immune organs and central nervous tissues. All these findings highlight 2′-DI as a potential prophylactic candidate for MS, with its capacity to restore immune homeostasis and alleviate neuroinflammation-driven neurodegeneration speculated to be potentially associated with increased AMPK phosphorylation signaling.

Increasing evidence highlights the pivotal role of energy metabolism in the immunopathogenesis of MS. For instance, propionic acid, a key metabolite derived from glycolysis and fatty acid metabolism, has been shown to reverse the Treg/Th17 cell imbalance, a hallmark of MS immunopathology (Duscha et al., 2020). Notably, 2′-DI, a crucial nucleoside component of the nucleoside metabolic pathway that involves the energy metabolism process, exerts regulatory effects on MS-related immune dysregulation. As a key regulator of lipid metabolism, glucose metabolism, and autophagy, AMPK acts to restore energy homeostasis by promoting ATP-producing catabolic pathways while inhibiting energy-consuming processes (Mihaylova and Shaw, 2011; Steinberg and Hardie, 2023; Høyer-Hansen et al., 2007). Previous studies have demonstrated that AMPK signaling improves mitochondrial homeostasis and protects oligodendrocytes in MS (Largani et al., 2019), and it also safeguards the CNS under pathological conditions by maintaining energy balance (Paintlia et al., 2013). Consistent with these findings, our study identified that differentially abundant metabolites in MS patients were predominantly enriched in energy metabolic processes, including ether lipid metabolism, galactose metabolism, and glycerophospholipid metabolism. In addition, we demonstrated that 2′-DI-mediated immune balance regulation is potentially correlated with AMPK phosphorylation upregulation, providing robust evidence for the involvement of energy metabolism in the preventive immunoregulation of MS. Notably, there may be another molecular mechanism by which 2′-DI is involved in directly regulating the energy metabolism pathway in MS progression, which needs further research validation.

Our study also revealed distinct regulatory effects of 2′-DI on T-cell subsets across different tissues. Notably, no significant differences in total CD8^+^ T-cell counts were observed in the brain, spleen, or peripheral blood of 2′-DI-treated mice compared with the vehicle controls. This finding suggests that 2′-DI primarily exerts direct regulatory effects on CD4^+^ T cells, whereas the observed reduction in IFN-γ+ CD8^+^ T-cell populations may be an indirect consequence of CD4^+^ T-cell modulation. In contrast, CD8^+^ T-cell numbers were slightly increased in the spinal cord, which may reflect a compensatory physiological response. This hypothesis warrants further experimental investigation to clarify the underlying mechanisms.

Our results revealed distinct tissue-specific alterations in lymphocyte distribution following 2′-DI intervention, with decreased lymphocyte infiltration in the peripheral blood, spinal cord, and brain and increased lymphocyte abundance in the spleen. Given the current lack of direct functional evidence verifying lymphocyte trafficking dynamics, splenic lymphocyte retention, S1P pathway involvement, or BBB functional changes, the mechanistic basis for this altered immune cell distribution remains unconfirmed. Collectively, 2′-DI ameliorated MS progression primarily by restoring peripheral and central Th1/Th2 and Th17/Treg immune balance to constrain excessive proinflammatory responses during the early pre-clinical stage of MS. The reduced CNS inflammatory infiltration observed in 2′-DI-treated mice may be a secondary phenotypic change, and its potential association with lymphocyte trafficking regulation requires further mechanistic validation. Importantly, 2′-DI exhibited no direct reparative effects on demyelinated lesions or injured axons. The observed mitigation of myelin damage and neuronal loss in EAE mice may be attributed to suppressed inflammatory infiltration rather than direct neural protection by 2′-DI. Owing to its robust immunomodulatory activity, 2′-DI may also provide prophylactic and therapeutic potential for other autoimmune diseases with similar immune dysregulation features, such as rheumatoid arthritis.

Our study has several limitations that should be acknowledged. First, the current study is a preliminary exploratory investigation with multiple experimental cohorts featuring relatively small sample sizes. Second, the metabolomic cohort enrolled a limited number of participants with incomplete clinical data, such as lack of active MRI lesions, glucocorticoid usage and disease-modifying therapy regimens, which may introduce confounding factors. Third, the safety profile of 2′-DI, such as systemic toxicity and long-term tolerability, remains lacking systematic evaluation. These factors are critical for the translation of 2′-DI into clinical practice and require thorough investigation in future studies. While the single 1 mg/kg dose of 2′-DI applied herein effectively relieved neuroinflammation in EAE, it may not be the optimal therapeutic dosage. Future dose-gradient studies will therefore identify the optimal safe and effective dose tailored to the EAE model. Fourth, the direct protective role of 2′-DI in myelin integrity preservation and myelin repair, a core pathological process in MS, remains to be further verified. Our current data do not demonstrate intrinsic myelin-repairing or axon-protective functions of 2′-DI, and the alleviated demyelination observed in EAE mice receiving 2′-DI is merely a secondary outcome of suppressed inflammatory infiltration. Myelin injury is major contributor to neurodegeneration and long-term disability in MS (Abdelhak et al., 2026), so clarifying 2′-DI’s effects on these processes will enhance our understanding of its therapeutic potential. Moreover, the detailed molecular crosstalk between 2′-DI and AMPK-mediated energy metabolism signaling pathways requires in-depth exploration. Elucidating these mechanisms will provide a more comprehensive understanding of how 2′-DI modulates immune homeostasis and neuroinflammation, which may facilitate the development of more effective preventive and therapeutic strategies targeting MS. Most importantly, this study fails to establish definitive causal evidence for the core AMPK-related regulatory mechanism. We only identified a correlative link between 2′-DI-induced upregulation of AMPK phosphorylation and the restoration of Th1/Th2 and Th17/Treg balance, without loss-of-function assays to validate causal dependency. Follow-up functional experiments will be implemented to confirm the causal connection between AMPK signaling and the protective effects of 2′-DI against MS. Notably, the preventive efficacy of 2′-DI was verified in a prophylactic preclinical EAE model with intervention initiated before clinical disease onset, while its therapeutic effects against established MS requires further validation. Despite these limitations, our findings provide novel mechanistic insights into the involvement of 2′-DI in MS immunopathogenesis and identify 2′-DI as a promising prophylactic candidate for this debilitating disease.

In conclusion, this study identifies 2′-DI as a key immunomodulator in MS. Clinically, MS patients exhibit reduced peripheral circulating 2′-DI, which may serve as a candidate metabolic biomarker for this disease. In EAE mouse models, prophylactic exogenous 2′-DI supplementation attenuates disease progression and inflammation by reducing Th1/Th17 cells, increasing Treg/Th2 cells, and regulating pro/anti-inflammatory mediators. Mechanistically, the immunomodulatory effects of 2′-DI are potentially correlated with elevated AMPK phosphorylation rather than definitively dependent on this pathway. Collectively, 2′-DI may serve as a promising prophylactic candidate for MS by inhibiting central neuroinflammation and correcting peripheral and central Th1/Th2 and Th17/Treg immune imbalance, with its immunomodulatory effects potentially involving AMPK phosphorylation upregulation, thereby alleviating neuroinflammation and neurodegeneration.

## Data Availability

The datasets presented in this study can be found in online repositories. The metabolomics data supporting the findings of this study have been deposited in the NIH Common Fund’s National Metabolomics Data Repository (NMDR), the Metabolomics Workbench (Study ID: ST005186).
