# Supplementary material for: 2′-deoxyinosine ameliorates multiple sclerosis through modulating Th1/Th2 and Th17/Treg cell balance with potential involvement of AMPK phosphorylation
Source: Front Pharmacol. 2026 Sep 17;17:1837446. doi: 10.3389/fphar.2026.1837446 (PMC13627318; doi:10.3389/fphar.2026.1837446)
Supplement: Supplementary file 1 [file Supplementaryfile1.docx]

**Supplementary materials**

**Figure S1.** The representative gating strategy for all flow cytometric analyses.

**Figure S2.** The transcriptomic profiles of the control, 0.5 mM-DI, and 1 mM-DI groups. (A) Volcano plot of the changes in metabolites. (A) Volcano plot of the DEGs between the control group and the 0.5 mM-DI group (left) and between the control group and the 1 mM-DI group (right). (B) KEGG classification analysis of the control and 1 mM-DI groups.

**Figure S3.** Administration of 2’-DI inhibits the proliferation of lymphocytes and the secretion of proinflammatory cytokines. (A) CCK-8 analysis of lymphocytes after treatment with different doses of 2’-DI (n=3). (B) Mean fluorescence intensity analysis of lymphocytes treated with different doses of 2’-DI (n=5). (C) qRT-PCR analysis of IFN-γ expression (n=3). (D) qRT-PCR analysis of the expression of Foxp3 (n=3). The data are presented as the mean ± SD. ns = no significance, **p* < 0.05, ***p* < 0.01.

**Figure S4.** Administration of 2’-DI inhibits the proliferation of T-cell populations in the spinal cord of EAE mice (N=3). (A) Representative density plot and paragraph (B) for flow cytometry analysis of T-cell populations in the spinal cord of EAE mice treated with vehicle (VE) or 2’-DI (DI). The presented results are representative of experiments repeated at least three times. The data are presented as the mean ± SD. ns = no significance, **p* < 0.05, ***p* < 0.01.

**Figure S5.** Administration of 2’-DI inhibits the proliferation of T-cell populations in the brain of EAE mice (N=4). (A) Representative density plot and paragraph (B) for flow cytometry analysis of T-cell populations in the brain of EAE mice treated with vehicle (VE) or 2’-DI (DI). The presented results are representative of experiments repeated at least three times. The data are presented as the mean ± SD. ns = no significance, **p* < 0.05, ***p* < 0.01.

**Figure S6.** Administration of 2’-DI inhibits the proliferation of T-cell populations in the spleen and peripheral blood of EAE mice. (A) Representative density plot and paragraph (B) showing the flow cytometry analysis of T-cell populations in the spleen of EAE mice treated with vehicle or 2’-DI (n=4). (C) Representative density plot and paragraph (B) showing the flow cytometry analysis of T-cell populations in the peripheral blood of EAE mice treated with vehicle or 2’-DI (DI) (n=3). The presented results are representative of experiments repeated at least three times. The data are presented as the mean ± SD. ns = no significance, **p* < 0.05, ***p* < 0.01.
